# Effect of Golden Wanhong Ointment on wound microbiota in patients with diabetic foot ulcer

**DOI:** 10.3389/fcimb.2026.1820893

**Published:** 2026-08-06

**Authors:** Rong Qiu, Liyin Yang, Xinghong Li, Saiyin Li, Lihua Liu, Jiayan Che

**Affiliations:** 1 Yunnan University of Chinese Medicine, Kunming, China; 2 Yunnan Provincial Hospital of Traditional Chinese Medicine / The First Affiliated Hospital of Yunnan University of Chinese Medicine, Kunming, China

**Keywords:** 16S *rRNA* sequencing, diabetic foot ulcers, Jinwanhong ointment, microbiome, wound microbiota

## Abstract

**Objective:**

To investigate the regulatory effect of the hospital preparation Golden Wanhong Ointment on wound microbiota structure in patients with diabetic foot ulcer (DFU), to provide microbiological evidence for its traditional functions of “clearing heat and detoxifying, removing necrotic tissue and promoting granulation.”

**Methods:**

A total of 31 DFU patients were enrolled, and 62 wound secretion samples were collected before and after treatment. 16S *rRNA* gene V3–V4 region amplicon sequencing was performed to analyze changes in microbiota composition and diversity. Wound area, pain score (VAS), and inflammatory factors (IL-6, TNF-α) were measured before and after treatment.

**Results:**

Compared to pre-treatment, post-treatment assessments showed significantly reduced wound area, lower pain scores, and decreased levels of IL-6 and TNF-α (all P<0.001). Sequencing yielded 4,579,390 high-quality Clean Reads, with an average of 73,861 high-quality reads per sample. Microbiota analysis revealed a significant compositional remodeling. of wound microbiota structure after treatment: at the phylum level, the relative abundance of Proteobacteria decreased while Firmicutes increased; at the genus level, the abundances of *Prevotella* and *Escherichia-Shigella*, which are closely associated with infection, were significantly reduced.

**Conclusion:**

Golden Wanhong Ointment may promote wound healing by regulating DFU wound microbiota structure, inhibiting the abundance of key pathogenic bacteria, reducing local inflammatory response, and improving the wound microenvironment. This study provides a modern scientific interpretation of its traditional effects from a microbiological perspective.

## 1 Background

Diabetes mellitus is a common chronic metabolic disease and has become a major global public health issue. In 2021, there were 537 million diabetes patients worldwide, with approximately 140 million patients in China ranking first in the world, and the number of patients in China is expected to increase to 174 million by 2045 (Sun et al., 2022). Diabetic foot ulcer (DFU) is one of the most common and severe chronic complications of diabetes. Its pathological basis includes peripheral neuropathy and peripheral vascular disease, leading to reduced foot sensation, impaired blood supply, susceptibility to secondary infection after minor trauma, and formation of chronic non-healing wounds (Gu et al., 2024). DFU is characterized by “four highs”: high incidence rate, high recurrence rate, high disability rate, and high mortality rate (Zhang et al., 2020).The global incidence of DFU is approximately 15%–25%, while the incidence in China is 8.1%, with an amputation rate of about 5.1% and an annual mortality rate of 14.4%. Treatment costs account for approximately one-third of total diabetes-related medical expenses, imposing a substantial social and economic burden on patients’ families (Bakker et al., 2016; Armstrong et al., 2023; Endocrinology Branch of Chinese Medical Association, China Endocrine and Metabolic Diseases Specialty Alliance, 2024). Therefore, effective management of DFU wounds has become a key link in improving patient prognosis and reducing social burden.

Wound infection is the core reason for delayed DFU healing. Recent studies have found that DFU wounds are complex and dynamic microbial systems, containing aerobic bacteria, anaerobic bacteria, and facultative anaerobic bacteria (Gu et al., 2026). These microorganisms can trigger persistent inflammatory responses, impede wound healing, and even lead to sepsis (Zhou et al., 2018; Jiang, 2024). Modern medical research has confirmed that wound infection and microbiota dysbiosis are critical factors in the refractory nature of DFU (Li, 2024). Traditional Chinese medicine (TCM) has accumulated rich experience in understanding and treating such conditions. In traditional Chinese medicine, DFU falls under the categories of “gangrene of toe” or “gangrene” (Liu and Gao, 2025). The earliest relevant record can be found in the Lingshu·Yongju Pian, which described toe gangrene lesions as tuoju. The classic text noted that lesions presenting with erythema, dark discoloration and tissue necrosis indicated a fatal and incurable state; for persistent non-necrotic lesions, urgent amputation was mandated to prevent mortality (Anonymous, 2022; Zheng et al., 2023). The Jin Dynasty classic Zhenjiu Jiayi Jing formally defined tuoju as an independent disease entity. TCM theorizes that the pathogenesis of this disorder stems from the combined effects of qi deficiency, pathogenic factors and blood stasis (Huangfu, 2021). Accordingly, the primary therapeutic approaches include heat-clearing and detoxification, debridement of necrotic tissue, and facilitation of tissue regeneration and wound repair.

Golden Wanhong Ointment (also known as Zi lian Gao) is a hospital preparation of the First Affiliated Hospital of Yunnan University of Chinese Medicine (Yunnan Provincial Hospital of Traditional Chinese Medicine). It possesses the functions of clearing heat and detoxifying, removing necrotic tissue and promoting granulation, and is widely used clinically in the treatment of burns, pressure ulcers, postoperative wounds, and various chronic ulcers, showing good efficacy (Wu and Ye, 2012; Liu et al., 2014; He et al., 2017; He et al., 2021). Although its clinical efficacy in DFU has been demonstrated, its exact mechanism of action—particularly how it regulates the complex microbial community environment at the micro level—has not yet been fully elucidated (Liu, 2020). This study used 16S *rRNA* sequencing to analyze changes in DFU wound microbiota before and after Golden Wanhong Ointment intervention, aiming to provide modern biological evidence for its functions of “clearing heat and detoxifying, removing necrotic tissue and promoting granulation,” and to offer new targets for DFU treatment.

## 2 Materials and methods

### 2.1 Study subjects and sample collection

#### 2.1.1 Inclusion criteria

①Meeting the DFU diagnostic criteria in the Chinese Guidelines for the Diagnosis and Treatment of Diabetic Foot (Gu et al., 2024).

②Age 40–75 years.

③No use of antibiotics or topical antimicrobial agents in the past month.

#### 2.1.2 Exclusion criteria

①Presence of severe heart, liver, or kidney diseases.

②Wound complicated by fungal infection.

③Allergy to any component of Huangjin Wanhong Ointment.

④Pregnant or breastfeeding women.

#### 2.1.3 Sample collection

Thirty-one DFU patients (20 males, 11 females; mean age 62.03 ± 12.72 years) were enrolled. Secretions were collected from the wound center using sterile cotton swabs (rotated three times) before treatment (before the first dressing change) and two weeks after treatment (after the last dressing change), placed in sterile centrifuge tubes, and stored at -80 °C resulting in a total of 62 samples.

#### 2.1.4 Composition and source

**Figure 1:**
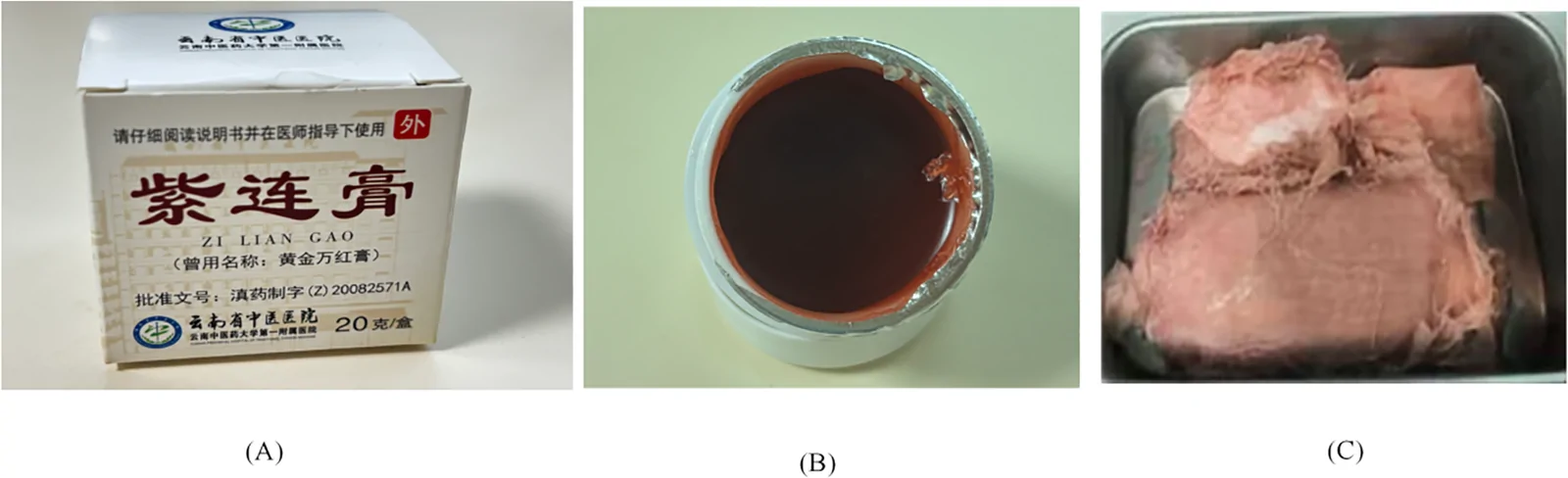
Golden Wanhong Ointment and the medicated gauze strip. **(A)** Outer packaging (approval number: Dian Yao Zhi (Z) 20082571A; 20 g/box), **(B)** The ointment itself, **(C)** GWO-impregnated gauze strip prepared under aseptic conditions.

Golden Wanhong Ointment is a topical formulation prepared exclusively by the First Affiliated Hospital of Yunnan University of Chinese Medicine (Yunnan Provincial Hospital of Traditional Chinese Medicine). It is an approved hospital formulation (approval number: Dian Yao Zhi Zi (Z) 20182571A; specification: 20 g per tube, see **Figure 1**). The main components include Lithospermum erythrorhizon, Coptis chinensis, Rehmannia glutinosa, Angelica sinensis, Scutellaria baicalensis, Polygonum cuspidatum, Sanguisorba officinalis, and Borneol, along with other auxiliary components. The ointment is prepared under strict quality control in the hospital pharmacy.

#### 2.1.5 Preparation of GWO-impregnated gauze strips

The dry gauze used for dressing changes was supplied sterile by the Central Sterile Supply Department (CSSD) of our hospital. The ointment was melted using a hot water bath, then homogeneously combined with the sterile gauze under aseptic conditions to form GWO-impregnated gauze strips. These strips were then sterilized using the dry heat method (160–170 °C for 2 hours), which is suitable for lipid-containing materials such as oil-impregnated gauze. After sterilization, the strips were stored under regulated conditions with a sterility validity period of seven days.

The wound dressing was changed once daily, regardless of wound condition. The inner layer consisted of GWO-impregnated gauze strips applied evenly over the wound surface, extending 3–5 cm beyond the wound margin; the number of gauze layers was adjusted to ensure full contact with the wound and even distribution of the ointment. The outer layer comprised six to eight layers of sterile gauze for additional protection. If exudate visibly penetrated the outermost gauze layer, the dressing was re-applied immediately.

#### 2.1.6 Treatment course

The total observation period was two weeks. After discharge, patients continued to undergo dressing changes during scheduled outpatient follow-up visits. Throughout the treatment period, standard systemic diabetes management was maintained to ensure glycemic stability.

#### 2.1.7 Collection of wound secretions for 16S sequencing

Wound secretions were collected before treatment and after two weeks of treatment with Golden Wanhong Ointment (day 1 was defined as the first day of ointment application, i.e., day 14). The wound was rinsed with 0.9% normal saline. If necessary, a moistened sterile gauze was used to gently wipe the wound surface. Sterile cotton swabs were then used to collect exudate or pus from the patient’s foot, and the swabs were placed into sterile tubes. The collected samples were immediately snap-frozen in liquid nitrogen and then transferred to a −80 °C freezer for storage. In the absence of a −80 °C freezer, samples could be stored at −20 °C for no more than one month, and repeated freeze-thaw cycles were avoided.

#### 2.1.8 Measurement of serum inflammatory cytokines

Fasting venous blood (2 mL) was collected from each patient before treatment and on day 14 after treatment. The blood samples were centrifuged to obtain the upper serum layer, which was then aliquoted and stored at −20 °C until analysis. Serum levels of tumor necrosis factor−α (TNF−α) and interleukin−6 (IL−6) were measured using enzyme−linked immunosorbent assay (ELISA).

#### 2.1.9 Ethical approval

This study was conducted in accordance with the Declaration of Helsinki and the Good Clinical Practice (GCP) guidelines. The study protocol (version 2.0, 2023.12.01) and informed consent form (version 2.0, 2023.12.01) were approved by the Ethics Committee of the First Affiliated Hospital of Yunnan University of Chinese Medicine/Yunnan Provincial Hospital of Traditional Chinese Medicine (initial review No.: YJYLL2023-010-01; re-review approval No.: YLL (2023) Ethics Review No. (010)-02; follow-up review No.: YLL2023-010-03). Written informed consent was obtained from all individual participants enrolled in the study.

### 2.2 Sequencing and data analysis

Total DNA was extracted from the 62 diabetic foot ulcer wound secretion samples using the CTAB method. The V3–V4 hypervariable region of the bacterial 16S *rRNA* gene was targeted for amplification using specific primers 341F (5’-CCTAYGGGRBGCASCAG-3’) and 806R (5’-GGACTACNNGGGTATCTAAT-3’) fused with sequencing adapters. PCR products were purified, quantified, and pooled to construct a sequencing library. Paired-end sequencing was performed on the Illumina NovaSeq 6000 platform to obtain raw reads. Raw data were first processed with Trimmomatic (v0.33) to filter low-quality bases and adapter sequences, and then with cutadapt (1.9.1) to remove primers, yielding Clean Reads. Subsequently, the DADA2 plugin in QIIME2 (2020.6) was used for denoising, including sequence correction, chimera removal, and merging paired-end reads, generating an amplicon sequence variant (ASV) feature table, achieving single-nucleotide resolution. Based on the Silva 138 database, a naive Bayes classifier combined with BLAST alignment was used for taxonomic annotation of ASV sequences, obtaining classification information from kingdom to species levels. Species abundance tables for each taxonomic level were constructed, and low-abundance features with abundance below 3 across all samples were filtered out.

### 2.3 Statistical analysis

SPSS.27.0 software was used for statistical analysis of wound area, IL-6, TNF-α, and pain scores before and after treatment. After testing for normality, data conforming to a normal distribution were analyzed using paired t-tests, while non-normally distributed data were analyzed using the Wilcoxon signed-rank test. P < 0.05 was considered statistically significant.

## 3 Results

### 3.1 Effect of Golden Wanhong Ointment on clinical indicators of DFU wounds

**Figure 2:**
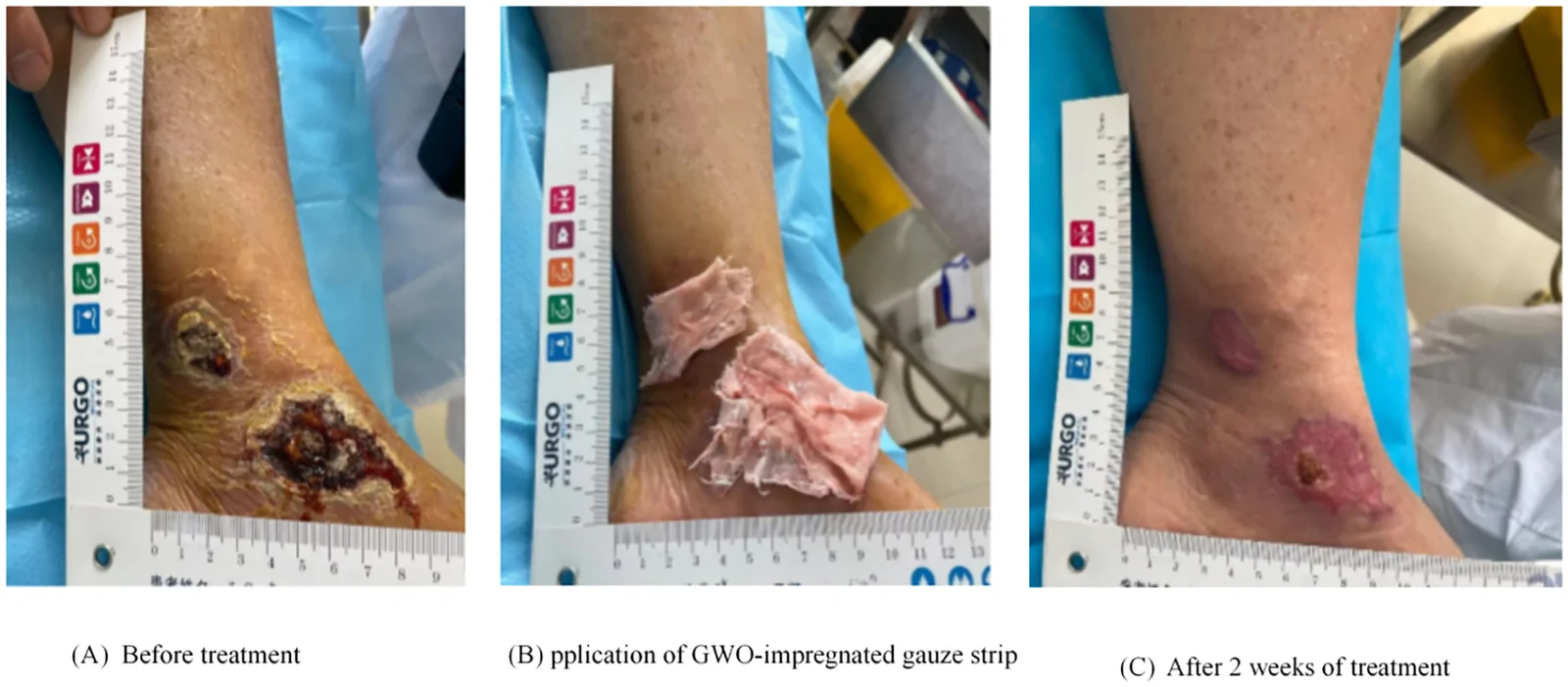
Clinical photographs of a representative patient. **(A)** Before treatment. **(B)** Dressing change with GWO-impregnated gauze strip. **(C)** After 2 weeks of treatment.

Among the 31 DFU patients included, there were 11 females and 20 males, with a mean age of 62.03 ± 12.72 years. As shown in **Table 1**, wound area, inflammatory factor levels, and pain scores were significantly improved after Golden Wanhong Ointment intervention as compared to before treatment. A comparison of the patient’s wound before and after treatment is presented in **Figure 2**.

**Table 1:** Comparison of clinical indicators before and after treatment (x¯ ± s, n=31).

Indicator	Before treatment	After treatment	*t*	*p*
Wound area (cm²)	30.25 ± 28.29	19.00 ± 18.93	5.609	<0.001
IL-6(pg/mL)	42.66 ± 14.66	39.14 ± 15.766	4.425	<0.001
TNF-α(pg/mL)	72.92 ± 35.60	67.53 ± 37.12	4.239	<0.001
Pain score (VAS)	5.68 ± 1.05	3.35 ± 1.05	14.84	<0.001

### 3.2 Sequencing data overview

A total of 5,064,268 paired-end raw reads were obtained. After quality control, 4,579,390 clean reads were retained, with sufficient sequencing depth for each sample.

### 3.3 Effect of Golden Wanhong Ointment on DFU wound microbiota

#### 3.3.1 Changes in microbiota composition at phylum, class, and order levels

**Figure 3:**
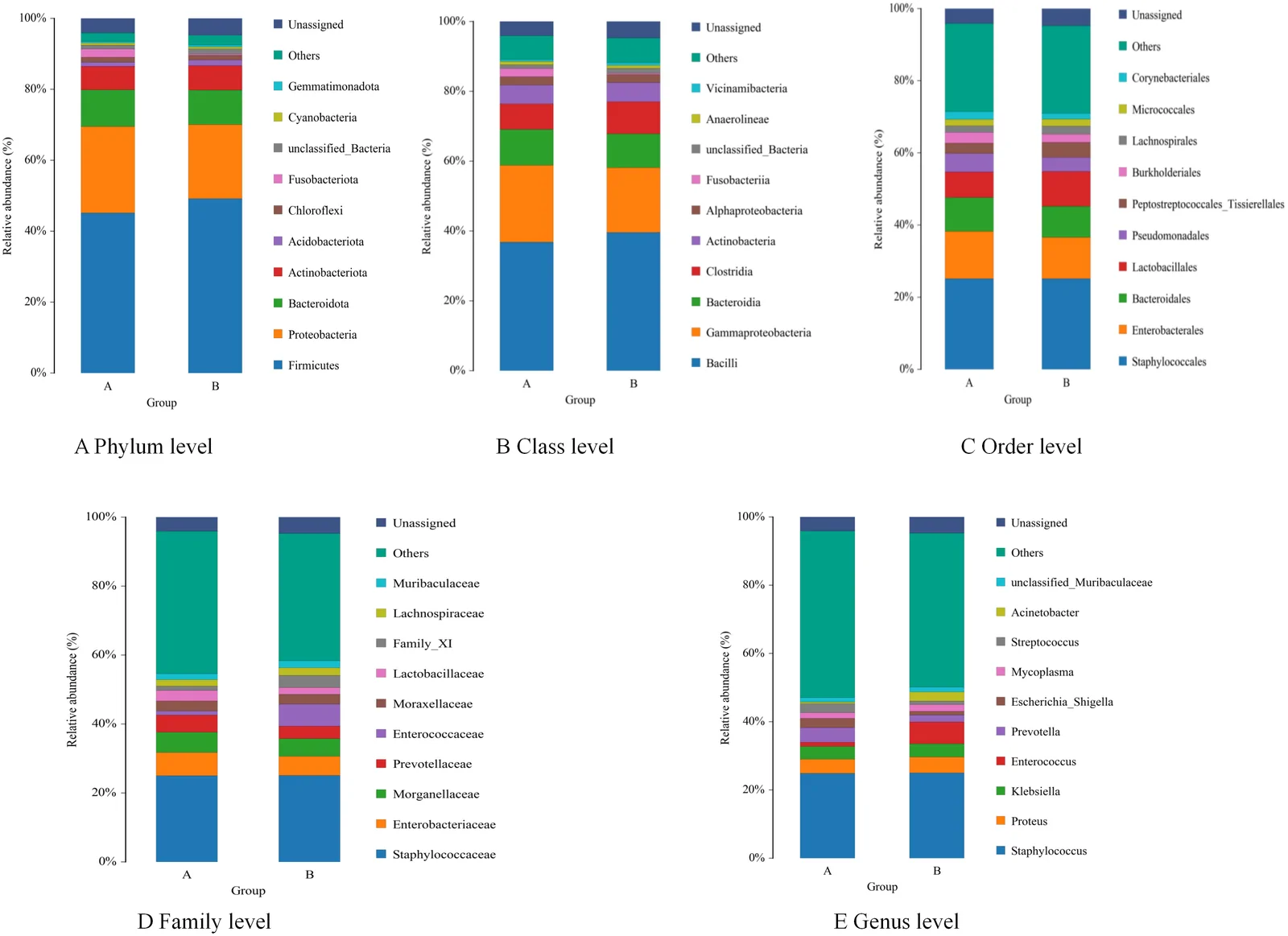
Compositional changes in the wound microbiota of diabetic foot ulcer patients before and after treatment with Golden Wanhong Ointment at five taxonomic levels. **(A)** Phylum level; **(B)** Class level; **(C)** Order level; **(D)** Family level; **(E)** Genus level. For each subpanel, Group A: before treatment (n=31); Group B: after two weeks of treatment (n=31). Bars indicate mean relative abundance. Only taxa with relative abundance >1% in at least one group are shown; the remainder are summarized as “Others”.

At the phylum level, the wound microbiota were dominated by Firmicutes and Proteobacteria both before and after treatment. After treatment, the relative abundance of Firmicutes showed an increasing trend, while that of Proteobacteria decreased. Bacteroidota and Actinobacteriota showed no significant changes (**Figure 3A**).

This study analyzed the composition of bacterial communities at three taxonomic levels: phylum, class, and order. At the phylum level, the wound microbiota were absolutely dominated by Firmicutes and Proteobacteria both before and after treatment. Quantitative comparison showed that the relative abundance of Firmicutes increased significantly after treatment compared with before treatment, while the relative abundance of Proteobacteria decreased correspondingly. The relative abundances of Bacteroidota and Actinobacteriota showed no significant differences between groups (**Figure 3A**). At the class level, the dominant classes before treatment were Bacilli (affiliated with Firmicutes) and Gammaproteobacteria (affiliated with Proteobacteria). After treatment, the relative abundance of Gammaproteobacteria decreased significantly, while that of Bacilli showed an increasing trend (**Figure 3B**). This trend was hierarchically consistent with the phylum-level results. Further analysis at the order level revealed (**Figure 3C**) that Staphylococcaceae was the core taxon with the highest relative abundance both before and after treatment. In addition, Enterobacterales, Bacteroidales, and Lactobacillales also accounted for high proportions. After intervention, the relative abundances of Staphylococcaceae and Lactobacillales, which are affiliated with Firmicutes, increased, while the abundance of Enterobacterales, which is affiliated with Proteobacteria, decreased. This indicates that the treatment intervention led to systematic changes in the wound microbiota structure, with the core characteristic being a general increase in the relative abundance of Firmicutes and their subordinate taxa, and a decrease in the abundance of Proteobacteria and their subordinate taxa.

#### 3.3.2 Key changes in microbiota composition at the family and genus levels

At the family level (**Figure 3D**), analysis showed that after treatment, the relative abundances of Enterobacteriaceae, Moraxellaceae, and Morganellaceae all exhibited a significant decreasing trend. In contrast, the relative abundance of Staphylococcaceae showed no consistent significant change, while that of Lactobacillaceae displayed an increasing trend.

At the genus level (**Figure 3E**), the analysis further revealed more specific structural changes in the microbiota. After treatment, the relative abundances of genera closely associated with infection that were dominant in pre-treatment samples, such as Klebsiella and Proteus, significantly decreased. Among these, the reductions in the abundances of *Prevotella* and *Escherichia-Shigella* were particularly pronounced. Conversely, the relative abundances of some genera, including Enterococcus, showed an increasing trend after the intervention.

This indicates that the therapeutic intervention not only altered the compositional patterns at higher taxonomic levels (phylum, class) but also triggered significant fluctuations in abundance at the specific family and genus levels. This was primarily characterized by a decrease in the abundance of a series of infection-related genera, accompanied by an increase in the abundance of certain other genera, collectively constituting a remodeling of the wound microbiota structure.

## 4 Discussion

This study demonstrated that after two weeks of intervention with Golden Wanhong Ointment, the wound area of patients with diabetic foot ulcer (DFU) was significantly reduced, pain scores decreased, and serum IL-6 and TNF-α levels were significantly lowered (P < 0.001). These results are consistent with previous clinical observations of Golden Wanhong Ointment in promoting chronic wound healing (Liu, 2020; Xu et al., 2025), confirming its effectiveness in alleviating local symptoms and accelerating wound repair. The reduction in wound area directly reflects the progression of tissue regeneration and epithelialization, while the decrease in pain scores suggests an alleviation of the local inflammatory response. Notably, a previous randomized controlled trial by our team demonstrated that Golden Wanhong Ointment was significantly superior to vaseline gauze in reducing DFU wound area and pain scores (both *P* < 0.05), indicating that the observed improvements are attributable to the ointment itself rather than spontaneous healing (Liu, 2020). To further explore its mechanism of action, this study employed 16S *rRNA* sequencing to analyze changes in the wound microbiota before and after treatment.

IL-6 and TNF-α are important inflammatory cytokines. As a pro-inflammatory cytokine, TNF-α not only directly mediates inflammatory injury but also induces the upregulation of IL-6 expression. IL-6 subsequently further amplifies the inflammatory signaling cascade, exacerbating tissue inflammatory pathological damage (Zheng and Guo, 2026). In the pathological process of DFU, persistently elevated inflammatory factors can directly damage tissue cells and impede wound healing by activating matrix metalloproteinases and inhibiting fibroblast function (Luo et al., 2024). The concurrent decrease in both factors in this study indicates that Golden Wanhong Ointment possesses a clear local anti-inflammatory effect, creating a favorable microenvironment for the wound to transition from a chronic inflammatory state to a reparative state. This finding is further supported by a study from our hospital showing that Golden Wanhong Ointment significantly reduced inflammatory cell infiltration in deep second-degree burn wounds (Xu et al., 2025).

16S *rRNA* sequencing revealed that the microbial community in DFU wounds was dysbiotic prior to treatment, with Firmicutes and Proteobacteria as the dominant phyla, consistent with previous reports (Li et al., 2024). Firmicutes encompass a diverse array of common wound pathogens, whereas Proteobacteria represent a major reservoir of Gram-negative pathogenic bacteria (Whitman, 2009). Collectively, these taxa form a pathogen-enriched microenvironment marked by low biodiversity and poor ecological resilience, which can severely impede wound healing by perpetuating local inflammation and facilitating biofilm formation (Patel et al., 2024). Following intervention with Golden Wanhong Ointment, the wound microbiota underwent substantial remodeling. At the phylum level, the relative abundance of Firmicutes increased, while that of Proteobacteria decreased. At the genus level, infection-associated genera such as *Prevotella* and *Escherichia-Shigella* were markedly reduced.

The difficulty in healing chronic wounds is often associated with persistent inflammation and biofilm formation (Falcone et al., 2021). During wound infection, bacteria are often embedded within a matrix of extracellular polymeric substances, forming complex microbial communities known as biofilms. Biofilms can protect bacteria from clearance by antibiotics and the host immune system, leading to persistent infection, with drug resistance potentially reaching thousands of times that of planktonic bacteria (Di Domenico et al., 2021; Das and Bal, 2024). *Prevotella*, a common anaerobic bacterium in wound infections, is prone to causing systemic infection and delayed healing under hypoxic conditions (Norimatsu and Ohno, 2020); its metabolites can activate the TLR2 pathway to induce TNF-α production (Bertelsen et al., 2020). The *Escherichia-Shigella* group is essentially a pathogenic variant of *Escherichia coli* with the ability to invade the host; its lipopolysaccharide (LPS) is a potent inflammatory activator that can directly stimulate macrophages to release IL-6 (Seferbekova et al., 2021; Xu et al., 2021). This study showed that Golden Wanhong Ointment effectively reduced the abundance of these two genera, accompanied by decreased levels of IL-6 and TNF-α. This suggests that Golden Wanhong Ointment may exert an indirect anti-inflammatory effect by inhibiting these key pathogenic bacteria and reducing sources of inflammatory stimuli.In addition to indirectly modulating the microbiota, the active ingredients in Golden Wanhong Ointment may also exert direct anti-inflammatory effects. Berberine and genistein, the main active components of Coptis chinensis (the sovereign drug in the formula), reduce the expression of pro-inflammatory factors such as TNF-α and IL-6 by inhibiting the NF-κB signaling pathway (Machha and Mustafa, 2005; Dhapola et al., 2024). Furthermore, berberine can disrupt cell membranes and cell walls, interfere with DNA replication and protein synthesis, and effectively inhibit or even destroy biofilms (Khan et al., 2022). Meanwhile, shikonin from *Lithospermum erythrorhizon* can reduce LPS-induced secretion of TNF-α and IL-6 by macrophages (Liu et al., 2025). In summary, the anti-inflammatory effect of Golden Wanhong Ointment represents a synergistic action of directly inhibiting inflammatory signaling pathways and indirectly reducing pathogen-induced inflammatory stimuli. This multi-target integrated regulatory model reflects the holistic regulatory characteristics of traditional Chinese medicine compound prescriptions. In conclusion, the anti-inflammatory mechanism of Golden Wanhong Ointment involves a dual pathway: on one hand, its active components intervene in inflammatory signaling pathways; on the other hand, it remodels the wound microbiota structure and reduces the abundance of key pathogenic bacteria like *Prevotella* and *Escherichia-Shigella*, thereby diminishing bacteria-derived inflammatory stimulation.

From the perspective of traditional Chinese medicine theory, Golden Wanhong Ointment possesses the functions of “clearing heat and detoxifying, removing necrotic tissue and promoting granulation.” This study found that its “clearing and resolving” effect is not manifested as broad-spectrum elimination of all bacteria, but rather specifically reduces the abundance of *Prevotella* and *Escherichia-Shigella*, which are closely associated with biofilm formation and chronic infection. “Removing necrotic tissue” may correspond to disintegrating the biofilms involving these genera and clearing chronic infectious foci; “promoting granulation” may stem from the inhibition of key pro-inflammatory genera, allowing the wound microenvironment to shift from a persistent inflammatory state towards repair, thereby promoting tissue regeneration. This study provides a modern scientific interpretation of this traditional efficacy from a microbiological perspective.

It is noteworthy that the results of this study showed that Golden Wanhong Ointment did not cause a general reduction in core pathogens such as *Staphylococcus* or *Klebsiella*, but rather manifested as a specific decrease in *Prevotella* and *Escherichia-Shigella*. This differs from the mode of action of some broad-spectrum antibiotics or antimicrobial dressings, suggesting that topical traditional Chinese medicine may work by regulating the ecological balance of the microbiota rather than through comprehensive elimination.

## 5 Conclusion

In summary, this study demonstrates that Golden Wanhong Ointment significantly remodels the wound microbiota of diabetic foot ulcers, characterized by a decreased abundance of infection-associated genera (*Prevotella* and *Escherichia-Shigella*) and a concurrent reduction in local inflammatory cytokines (IL-6 and TNF-α). These findings provide a modern microbiological interpretation of its traditional functions of “clearing heat, detoxifying, removing necrotic tissue, and promoting granulation.” However, the absence of a parallel control group and the limited sample size are key limitations; further validation in larger-scale controlled studies is warranted.

## Data Availability

The datasets presented in this article are not readily available because they contain identifiable information from human subjects that cannot be shared in open access repositories for legal and ethical reasons. Anonymized data will be shared upon reasonable request from any qualified investigator. Requests should be directed to the corresponding authors: LL (liulih781006@126.com) and JC (2279391826@qq.com).

## References

[B1] Anonymous (2022). Huangdi Neijing Lingshu (Guangzhou, China: Guangdong Science and Technology Press), Traditional Chinese Medicine Classic Palm Treasure Series, 1st edition, 232.

[B2] Armstrong D. G. Tan T. Boulton A. J. M. Bus S. A . (2023). Diabetic foot ulcers: a review. JAMA 330, 62–75. doi: 10.1001/jama.2023.10578 37395769 PMC10723802

[B3] Bakker K. Apelqvist J. Lipsky B. A. Van Netten J. J . (2016). The 2015 IWGDF guidance documents on prevention and management of foot problems in diabetes: development of an evidence-based global consensus. Diabetes Metab. Res. Rev. 32, 2–6. doi: 10.1002/dmrr.2694 26409930

[B4] Bertelsen A. Elborn J. S. Schock B. C. (2020). Infection with *Prevotella* nigrescens induces TLR2 signalling and low levels of p65 mediated inflammation in Cystic Fibrosis bronchial epithelial cells. J. Cyst Fibros 19, 211–218. doi: 10.1016/j.jcf.2019.09.005 31607634

[B5] Das I. J. Bal T. (2024). pH factors in chronic wound and pH-responsive polysaccharide-based hydrogel dressings. Int. J. Biol. Macromol. 279, 135118. doi: 10.1016/j.ijbiomac.2024.135118 39208902

[B6] Dhapola R. Beura S. K. Sharma P. Singh S. K. Harikrishnareddy D . (2024). Oxidative stress in Alzheimer's disease: current knowledge of signaling pathways and therapeutics. Mol. Biol. Rep. 51, 48. doi: 10.1007/s11033-023-09021-z 38165499

[B7] Di Domenico E. G. Marchesi F. Cavallo I. Toma L. Sivori F. Papa E. . (2021). The impact of bacterial biofilms on end-organ disease and mortality in patients with hematologic Malignancies developing a bloodstream infection. Microbiol. Spectr. 9, e55021. doi: 10.1128/Spectrum.00550-21 34406812 PMC8552682

[B8] Endocrinology Branch of Chinese Medical Association, China Endocrine and Metabolic Diseases Specialty Alliance (2024). Expert consensus on treatment of diabetic foot ulcers (2024). Chin. J. Endocrinol. Metab. 40, 565–569. doi: 10.3760/cma.j.cn311282-20240625-00281 30704229

[B9] Falcone M. De Angelis B. Pea F. Scalise A. Stefani S. Tasinato R. . (2021). Challenges in the management of chronic wound infections. J. Global Antimicrob. Resist. 26, 140–147. doi: 10.1016/j.jgar.2021.05.010 34144200

[B10] Gu Y. Chen Z. Zhang L. Wang S . (2026). Analysis of the distribution and drug resistance of common pathogenic bacteria on wound surfaces in patients with diabetic foot ulcers. China J. Pathogen. Biol. 21, 841–844. doi: 10.13350/j.cjpb.260624

[B11] Gu Y. Ran X. Guo L. Guo J. Ju S. Cao Y . (2024). China guidelines for the diagnosis and treatment of diabetic foot. Chin. J. Clin. Physicians 52, 1287–1296. doi: 10.4103/vit.vit_15_19

[B12] He W. Yue J. Wang Y. Zhang Y. Dong Y. Zhang M. . (2021). Clinical observation on the application of the traditional Chinese medicine preparation Huangjinwanhong Gao in preventing and treating pressure ulcers caused by surgical positions. China Community Physician 37, 91–92.

[B13] He W. Zhang Y. Wang Y. Wang J . (2017). Observation on the application of Huangjin Wanhong Ointment in improving skin conditions after posterior approach PKP surgery in elderly patients. Inner Mongolia J. Traditional Chin. Med. 36, 85. doi: 10.16040/j.cnki.cn15-1101.2017.15.075

[B14] Huangfu M. (2021). Acupuncture and Moxibustion of the Jia and Yi Meridians (Beijing: Publishing Group Digital Media Co., Ltd., 1st edition), 144.

[B15] Jiang H. (2024). “ Investigating the mechanism of sodium alginate hydrogel loaded with arginine-loaded silver nanoparticles in promoting diabetic wound healing,” in Dalian Medical University. Dalian: Dalian Medical University. doi: 10.26994/d.cnki.gdlyu.2024.000071

[B16] Khan S. Hussain A. Attar F. Bloukh S. H. Edis Z. Sharifi M. . (2022). A review of the berberine natural polysaccharide nanostructures as potential anticancer and antibacterial agents. Biomedicine Pharmacotherapy 146, 112531. doi: 10.1016/j.biopha.2021.112531 34906771

[B17] Li Y. (2024). “ Comparison of microbial community distribution in diabetic foot wounds at different stages,” in Shanxi Medical University. Taiyuan: Shanxi Medical University.

[B18] Li Y. Zhang L. He M. Zhao Y . (2024). Sequence analysis of microbiota in clinical human cases with diabetic foot ulcers from China. Heliyon 10, e34368. doi: 10.1016/j.heliyon.2024.e34368 39104504 PMC11298921

[B19] Liu G. Gao H. (2025). Expert consensus on traditional Chinese medicine external treatment methods for diabetic foot ulcers. World J. Traditional Chin. Med. 20, 1442–1449.

[B20] Liu M. (2020). “ Clinical efficacy observation of external application of Huangjin Wanhong Ointment in treating diabetic foot ulcers with damp-heat toxin excess syndrome,” in Yunnan University of Traditional Chinese Medicine. Kunming: Yunnan University of Chinese Medicine 1. doi: 10.27460/d.cnki.gyzyc.2020.000057

[B21] Liu Y. He W. Shi P. Nie J. Zhang Y. Huang H . (2014). Clinical observation of 75 cases on the efficacy of Huangjin Wanhong Ointment in preventing skin injuries at pressure points of pneumatic tourniquets. China Ethnic Folk Med. 23, 51–52.

[B22] Liu Z. Wang J. Peng J . (2025). Research progress on the regulation of the adenylate kinase pathway by traditional Chinese medicine monomers in the prevention and treatment of sepsis. World J. Traditional Chin. Med. 20, 327–335. doi: 10.5539/mas.v2n5p127

[B23] Luo C. Akhtar M. Min W. Bai X. Ma T. Liu C . (2024). Domain of unknown function (DUF) proteins in plants: function and perspective. Protoplasma 261, 397–410. doi: 10.1007/s00709-023-01917-8 38158398

[B24] Machha A. Mustafa M. R. (2005). Chronic treatment with flavonoids prevents endothelial dysfunction in spontaneously hypertensive rat aorta. J. Cardiovasc. Pharmacol. 46, 36–40. doi: 10.1097/01.fjc.0000162769.83324.c1 15965352

[B25] Norimatsu Y. Ohno Y. (2020). First report of subcutaneous abscess caused by Porphyromonas gingivalis. IDCases 21, e917. doi: 10.1016/j.idcr.2020.e00917 32760652 PMC7390846

[B26] Patel M. Patel V. Shah U. Patel A . (2024). Molecular pathology and therapeutics of the diabetic foot ulcer; comprehensive reviews. Arch. Physiol. Biochem. 130, 591–598. doi: 10.1080/13813455.2023.2219863 37294861

[B27] Seferbekova Z. Zabelkin A. Yakovleva Y. Afasizhev R. Dranenko N. O. Alexeev N. . (2021). High rates of genome rearrangements and pathogenicity of Shigella spp. Front. Microbiol. 12, 628622. doi: 10.3389/fmicb.2021.628622 33912145 PMC8072062

[B28] Sun H. Saeedi P. Karuranga S. Pinkepank M. Ogurtsova K. Duncan B. B. . (2022). IDF Diabetes Atlas: global, regional and country-level diabetes prevalence estimates for 2021 and projections for 2045. Diabetes Res. Clin. Pract. 183, 109119. doi: 10.1016/j.diabres.2021.109119 34879977 PMC11057359

[B29] Whitman W. B. E. (2009). Systematic Bacteriology. New York, NY: Springer. doi: 10.1007/978-0-387-68489-5

[B30] Wu H. Ye J. (2012). Clinical observation of the formulation Huangjin Wan Hong Ointment in treating facial herpes simplex. Yunnan J. Traditional Chin. Med. 33, 42–43. doi: 10.16254/j.cnki.53-1120/r.2012.04.010

[B31] Xu L. Cai Z. Xu Y. Liu L. He W. Zhong X . (2025). Study on the reparative effects and mechanisms of Huangjin Wanhong Ointment on deep second-degree burn wounds in SD rats. Yunnan Med. J. 46, 80–83. doi: 10.20282/j.cnki.1006-4141.2025.01.22

[B32] Xu J. Zhang R. Yu X. Zhang X. Liu G. Liu X . (2021). Molecular characteristics of novel phage vB_ShiP-A7 infecting multidrug-resistant Shigella flexneri and Escherichia coli, and its bactericidal effect *in vitro* and *in vivo*. Front. Microbiol. 12, 698962. doi: 10.3389/fmicb.2021.698962 34512574 PMC8427288

[B33] Zhang Y. Lazzarini P. A. McPhail S. M. Van Netten J. J. Armstrong D. G. Pacella R. E . (2020). Global disability burdens of diabetes-related lower-extremity complications in 1990 and 2016. Diabetes Care 43, 964–974. doi: 10.2337/dc19-1614 32139380

[B34] Zheng C. Guo W. (2026). The effect of arctiin on cognitive function in rat models of post-traumatic stress disorder through modulation of the PI3K/AKT signaling pathway. China J. Immunol. 42, 316–321.

[B35] Zheng Y. Wu S. Cai M. Y. Su H. Wu H. Zhou M. . (2023). Analysis of acupuncture therapy for abscesses and ulcers in the Huangdi Neijing. J. Anhui Univ. Chin. Med. 42, 38–40.

[B36] Zhou J. Yao D. Qian Z. Hou S. Li L. Jenkins A. T. A. . (2018). Bacteria-responsive intelligent wound dressing: simultaneous in situ detection and inhibition of bacterial infection for accelerated wound healing. Biomaterials 161, 11–23. doi: 10.1016/j.biomaterials.2018.01.024 29421548

